# Brain Shape Changes Associated With Cerebral Atrophy in Healthy Aging and Alzheimer’s Disease

**DOI:** 10.3389/fmech.2021.705653

**Published:** 2021-07-19

**Authors:** Yana Blinkouskaya, Johannes Weickenmeier

**Affiliations:** Department of Mechanical Engineering, Stevens Institute of Technology, Hoboken, NJ, United States

**Keywords:** cerebral atrophy, brain aging, Alzheimer’s disease, multiphysics modeling, finite element modeling, brain shape changes

## Abstract

Both healthy and pathological brain aging are characterized by various degrees of cognitive decline that strongly correlate with morphological changes referred to as cerebral atrophy. These hallmark morphological changes include cortical thinning, white and gray matter volume loss, ventricular enlargement, and loss of gyrification all caused by a myriad of subcellular and cellular aging processes. While the biology of brain aging has been investigated extensively, the mechanics of brain aging remains vastly understudied. Here, we propose a multiphysics model that couples tissue atrophy and Alzheimer’s disease biomarker progression. We adopt the multiplicative split of the deformation gradient into a shrinking and an elastic part. We model atrophy as region-specific isotropic shrinking and differentiate between a constant, tissue-dependent atrophy rate in healthy aging, and an atrophy rate in Alzheimer’s disease that is proportional to the local biomarker concentration. Our finite element modeling approach delivers a computational framework to systematically study the spatiotemporal progression of cerebral atrophy and its regional effect on brain shape. We verify our results *via* comparison with cross-sectional medical imaging studies that reveal persistent age-related atrophy patterns. Our long-term goal is to develop a diagnostic tool able to differentiate between healthy and accelerated aging, typically observed in Alzheimer’s disease and related dementias, in order to allow for earlier and more effective interventions.

## 1 INTRODUCTION

**FIGURE 1 |:**
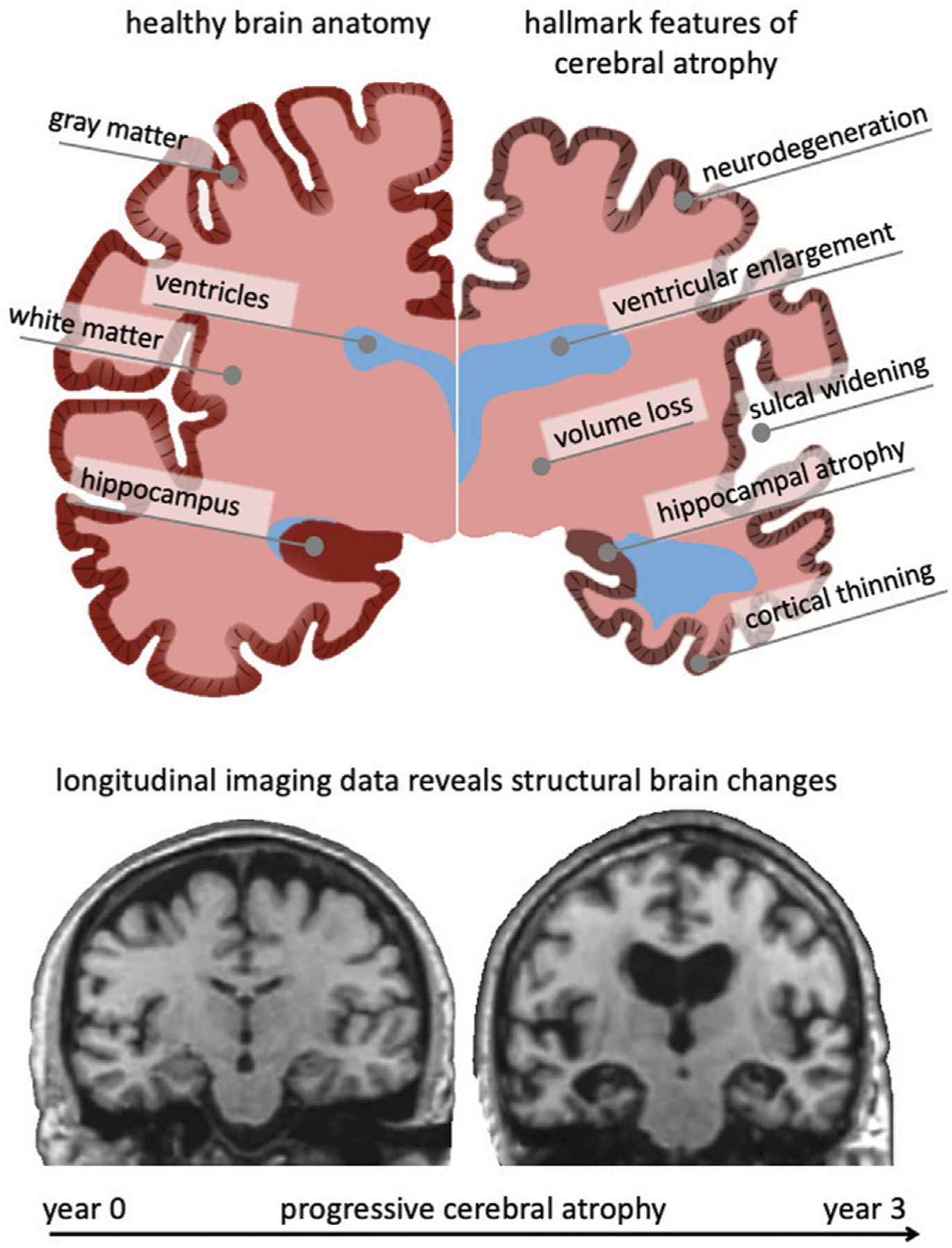
The aging brain undergoes cerebral atrophy which describes the morphological shape changes observed in both healthy and pathological aging. They include neurodegeneration, cortical thinning, volume loss, white matter degeneration, sulcal widening, and ventricular enlargement. As we age, subcellular and cellular aging mechanisms gradually result in these organ-level changes that are visible in cross-sectional imaging studies. Gradually growing availability of longitudinal data provides new insight into progressive brain deterioration over several years and allows to quantify personalized progression of brain aging, underlying pathology, and its cognitive impact. Here, we show two coronal slices of a subject with severe Alzheimer’s disease from the Alzheimer’s disease Neuroimaging Initiative, that highlight their significant atrophy during a 3-year period.

Brain aging is characterized by a myriad of biological, chemical, and mechanical hallmark features. While biological and chemical aging processes have been studied for decades, the mechanical aspects of brain aging remain understudied (Raz and Rodrigue, 2006; Hall et al., 2020). The brain undergoes several key morphological changes referred to as cerebral atrophy which manifests primarily as gray and white matter volume loss, ventricular enlargement, and sulcal widening (Fjell and Walhovd, 2010). While healthy brain aging is characterized by these changes, neurodegenerative diseases, such as Alzheimer’s disease (AD) and related dementias, exhibit a significant acceleration of brain aging mechanisms that cause a noticeable divergence from the healthy atrophy trajectory observed in cross-sectional studies (Coupé et al., 2019). Figure 1 shows a qualitative comparison between a healthy brain (left hemisphere) and a brain exhibiting severe age-related atrophy features (right hemisphere). Strikingly, the changes in the aging brain become so pervasive that they are clearly visible in medical images (Lockhart and DeCarli, 2014). Despite each person’s brain looking differently, cross-sectional imaging studies reveal significant trends in volume loss, ventricular enlargement, cortical thinning, and the emergence of white matter lesions (Walhovd et al., 20112011; Suzuki et al., 2019).

Brain aging is a highly heterogeneous process that is strongly linked to local cellular composition as well as the gradual aggregation of neurotoxic proteins and waste products that fail to drain into the glymphatic system (Boland et al., 2018). The superposition of metabolic slowing and decreased cellular regeneration in most of the brain, leads to structural and functional degeneration that drives cognitive decline (Ownby, 2010; Mattson and Arumugam, 2018). AD is characterized by the accumulation of neurotoxic amyloid beta plaques that interfere with normal synaptic transmission (Reddy and Beal, 2008; Milà-Alomà et al., 2020) and neurofibrillary tangles that disrupt axonal transport causing loss of signal transmission and axon death (Malpetti et al., 2020). Both proteins exhibit a prion-like behavior in that they recruit healthy protein, trigger their misfolding, and gradually form growing plaques and tangles (Jack and Holtzman, 2013). This leads to their systematic spread throughout the brain (Jack et al., 2013). While plaques spread extracellularly, tangles spread primarily along the structural axonal network and are able to eventually reach distant brain regions (Kim et al., 2019). This systemic infiltration of the brain has major implications for brain function such as memory, motor control, behavior, and ultimately death (Mattson, 2004).

From a mechanics perspective, brain aging is drastically understudied as it may provide new avenues to broaden our understanding of the relationship between cell- and tissue-level neurodegeneration and their aggregated effect on organ level morphological shape changes (Hall et al., 2020). Only a few studies have presented a mechanistic model of cerebral atrophy and are based on either non-rigid registration of two medical images (Karaçali and Davatzikos, 2006; Khanal et al., 2017) or the finite element method (Camara et al., 2006; Weickenmeier et al., 2018; Harris et al., 2019; Schäfer et al., 2019). Registration methods aim at minimizing intensity differences between two images by iteratively distorting a moving image to match the reference image. This minimization process may be subject to elasticity constraints derived from mechanics (Hamamci and Unal, 2013; Garcia et al., 2018). Finite element-based approaches are based on a constitutive model of volume loss that is implemented for two or three dimensional simulations (Budday and Kuhl, 2020). Harris et al. developed a two dimensional sagittal and coronal brain model to simulate volume loss representative for the brain’s response following a traumatic brain injury (Harris et al., 2019). The model is calibrated such that gray matter (GM) and white matter (WM) undergo different atrophy rates and shows an overall contraction of the cross-sectional brain image. The model does not capture aging-related ventricular enlargement, most likely due to the boundary conditions imposed on the model at the inferior edge of the brainstem. In a similar approach, Schäfer et al. presented a multiphysics model that couples protein spread in AD and volume loss (Schäfer et al., 2019). The model incorporates anisotropic diffusion of intracellular tau protein along the axon network. The two dimensional finite element (FE) model is characterized by an overall uniform area shrinking, although ventricular area marginally increases and cortical folds remain close together. In order to use computational modeling as a diagnostic tool to differentiate between healthy and pathological aging, simulation accuracy hast to be improved.

Here, we expand on a multiphysics model of cerebral atrophy which allows to differentiate between healthy and pathological aging (Weickenmeier et al., 2018; Schäfer et al., 2019). We employ classical continuum theory and model cerebral atrophy as negative growth *via* a multiplicative split of the deformation gradient into an atrophy part and an elastic part (Schäfer et al., 2019). Accelerated aging is driven by the gradual accumulation of an AD biomarker. We assume the atrophy factor to increase proportional to the biomarker concentration which we diffuse in the brain *via* a reaction-diffusion model, see **Section 2**. Using a subject specific FE model, we simulate healthy and AD-related brain aging and compare our model’s response to cross-sectional data reported in literature. Our comparison focuses on the hallmark features of cerebral atrophy and shows good qualitative agreement with the persistent trends observed in large-scale imaging studies.

## 2 METHODS

### 2.1 Multiphysics Model of Cerebral Atrophy

Our goal is to identify differences in spatiotemporal atrophy patterns characteristic for healthy and AD-related brain aging. Therefore, we formulate a multiphysics approach that couples mechanics-driven volume loss and the biology-driven spreading of toxic proteins (Weickenmeier et al., 2018). In our constitutive model, we pose that healthy aging is linked to a steady volume loss in gray and white matter tissues, while AD accelerates atrophy proportional to the local toxic protein level (Schäfer et al., 2019). We solve our continuum problem on an anatomically accurate finite element (FE) brain model and quantify hallmark features of cerebral atrophy including volume loss, cortical thinning, ventricular enlargement, and sulcal widening.

#### 2.1.1 Continuum Model for Protein Spread

AD is characterized by the accumulation and spreading of misfolded, neurotoxic proteins (Jucker and Walker, 2018). Post-mortem studies on AD patients have shown that protein spread follows a characteristic spatial pattern that is characterized by consistent onset locations and spreading pathways (Jack et al., 2013). Mathematically, these progression patterns are well approximated by a reaction-diffusion model known as the Fisher-Kolmogorov equation (Fisher, 1937; Kolmogorov et al., 1937). We define the concentration of misfolded protein, c, that spreads *via* linear diffusion.

(1) $$\frac{\partial c}{\partial t}=d\;\text{Δ}c+\alpha \;c\;[1-c],$$

where *d* is the isotropic diffusion constant, Δ *c* denotes the Laplacian of the protein concentration *c*, and *α* controls the growth rate of the concentration. For a derivation of the kinetic equations governing the prion-like behavior of proteins linked to AD, we refer the reader to our previous works (Schäfer et al., 2019; Weickenmeier et al., 2019). In brief, we derive a kinetic model that accounts for two configurations of the protein, a healthy state and a misfolded state. We then derive a kinetic equation that balances the total amount of healthy protein *p* and misfolded protein $\tilde{p}$, as a function of production rate of healthy protein *k*_0_, clearance rate of healthy and misfolded proteins *k*_1_ *k*_1_, and conversion of healthy to misfolded protein *k*_12_.

(2) $$\frac{\partial p}{\partial t}=k_{0}-k_{1}\;p-k_{12}p\tilde{p}\text{ and }\frac{\partial \tilde{p}}{\partial t}=-{\tilde{k}}_{1}\tilde{p}+k_{12}p\tilde{p}.$$

Through introduction of the misfolded protein concentration *c*, which may vary between 0 and 1, equilibrium considerations, and re-parameterization of the governing Eq. 2, we arrive at the partial differential Eq. 1, with

(3) $$\alpha =k_{12}\frac{k_{0}}{k_{1}}-{\tilde{k}}_{\cdot 1}.$$

Model parameters *d* and α allow to adjust for the amount of spread and progression speed of misfolded proteins observed in individual subjects affected by varying AD severity.

#### 2.1.2 Continuum Model for Cerebral Atrophy

To model the mechanical behavior of the brain, we use the nonlinear equations of continuum theory and introduce the mapping ***φ*** from the undeformed, unloaded configuration $B_{0}$ at time t_0_ to the deformed, loaded configuration $B_{t}$ at time t. We adopt the conventional notation, **x** = ***φ***(**X**, *t*), where $x\in B_{t}$ denotes the position vector in the deformed configuration at time *t* and $X\in B_{0}$ denotes the position vector of the initial configuration at time *t*_0_. We characterize local deformations by introducing the deformation gradient, **F**(**X**, *t*) = ∇_*X*_***φ*** (**X**, *t*) and local volume changes by its determinant, *J* = det (**F**). Following previous work, we model cerebral atrophy as volumetric shrinking and use the classical approach of splitting the deformation gradient into an elastic part **F**^*e*^ and an atrophy part **F**^*a*^ (Schäfer et al., 2019). The multiplicative decomposition of the deformation gradient, **F** = ∇_*X*_***φ***, yields

(4) $$F=F^{e}\cdot F^{a}\text{ with }J=J^{e}J^{a}.$$

The multiplicative split extends to the Jacobian *J* which breaks down into an elastic volume change *J*^*e*^ = det(**F**^*e*^) and volume loss by cerebral atrophy *J*^*a*^ = det(**F**^*a*^). To characterize the hyperelastic material behavior of brain tissue, we adopt the neo-Hookean strain energy density function Ψ_0_ as the atrophy-weighted elastic stored energy Ψ, which depends exclusively on the elastic part of the deformation gradient,

(5) $${\text{Ψ}}_{0}=J^{a}\text{Ψ},\text{ with Ψ}=\frac{1}{2}\mu \left[F^{e}:F^{e}-3-2\ln\left(J^{e}\right)\right]+\frac{1}{2}\lambda \ln^{2}\left(J^{e}\right).$$

Parameters *μ* and *λ* are the standard Lamé coefficients which can be expressed *via* Young’s modulus *E* and the Poisson’s ratio *ν* in the elastic limit as *λ* = *Eν*/[[1 + *ν*][1 − 2*ν*]] and *μ* = *E*/[2[1 + *ν*]. Following arguments of thermodynamics, we can derive the first Piola-Kirchhoff stress tensor **P**,

(6) $$P=\frac{\text{d}\psi_{0}}{\text{d}F}=J^{\text{a}}\frac{\text{d}\psi }{\text{d}F^{e}}=J^{a}\left[\mu F^{\text{e}}+\left[\lambda \ln\left(J^{\text{e}}\right)-\mu \right]F^{\text{eT}}\right].$$

The Piola-Kirchhoff stress tensor is governed by the quasistatic balance of linear momentum,

(7) $$0=\text{Div}\;(P)+F^{\varphi }\text{ in Ω,}$$

where Ω denotes the domain which is the brain. We assume that we can neglect external body forces **F**^*φ*^ = 0. In our multiphysics framework here, the atrophy problem is coupled to the protein spreading problem through the atrophy part of the deformation gradient **F**^*a*^, which is considered to be a function of age and biomarker concentration *c*. More specifically, we assume that gray and white matter atrophy is purely isotropic,

(8) $$F^{a}=\sqrt[{3}]{\vartheta }I\text{ and }F^{e}=\frac{F}{\sqrt[{3}]{\vartheta }}\text{, }$$

where we introduced a measure for volume loss *ϑ* which is related to cerebral atrophy *J*^*a*^,

(9) $$\vartheta =J^{a}\text{ and }J^{e}=\frac{J}{\vartheta }\text{. }$$

We propose a constitutive model for the evolution of the atrophy measure *ϑ* that allows to differentiate between healthy brain aging and accelerated aging observed in many neurodegenerative diseases such as AD (Weickenmeier et al., 2018; Schäfer et al., 2019). As such, we introduce a health atrophy rate, *G*_*h*_, as well as a biomarker concentration, c, dependent atrophy rate, *G*_*c*_, which allows us to capture accelerated cerebral atrophy due to the progressive accumulation of misfolded, neurotoxic protein. Our model is formulated such that natural atrophy is accelerated if the biomarker concentration, *c*, exceeds a critical threshold, *c*^crit^, such that the evolution equation reads.

(10) $$\dot{\vartheta }=[1+\gamma (c)]G_{h}=\{\begin{array}{ll} G_{h} & \text{if }c<c^{\text{crit }}\\ G_{h}+G_{c} & \text{if }c\geq c^{\text{crit }} \end{array},\;\text{where }\gamma (c)=\frac{G_{c}}{G_{h}}H\left(c-c^{\text{crit }}\right)\text{. }$$

Here, $H\left(c-c^{\text{crit}}\right)$ denotes the Heaviside step function and marks the transition from healthy to accelerated, or diseased, atrophy at *c*^crit^. Healthy and diseased atrophy rates, *G*_*h*_ and *G*_*c*_, may be treated as subject-specific aging parameters that can be tuned to capture their specific progression behavior.

### 2.2 Finite Element Implementation

We implemented our continuum model in the finite element software Abaqus (Simulia, Providence RI) and solved our coupled problem as a thermo-mechanical analysis. We add the nonlinear source term of the protein spreading equation (Raz and Rodrigue, 2006) to the standard heat transfer problem using the subroutine HETVAL which requires the flux, *f*^*c*^= *α c* [1 – *c*] and rate of change of heat flux per temperature, d*f*^*c*^/d*c* = *α*[1 − 2*c*]*.* Similarly, we incorporate our constitutive material model using the user subroutine UMAT which requires Cauchy stress and its Jaumann rate. To determine Cauchy stress at the integration point level, we calculate the atrophy factor *via* a finite difference scheme,

(11) $$\dot{\vartheta }=\frac{\vartheta -\vartheta_{n}}{\text{Δ}t},\text{ such that }\vartheta =\vartheta_{n}+[1+\gamma (c)]G_{h}\text{Δ}t,$$

where (∘) and (∘)_*n*_ denote the unknown quantity at *t* = *t*_*n*+1_ and the converged quantity at the previous time step *t* = *t*_*n*_, respectively, and Δ*t* = *t* − *t*_*n*_ > 0 is the current time increment. Here, we approximate the Heaviside step function H in *γ*(*c*) Eq. 10) as a smooth function,

(12) $$H\left(c-c^{\text{crit}}\right)=\frac{1}{1+\exp\left(\beta \left(c-c^{\text{crit }}\right)\right)},$$

where *β* controls the transition between the two states. We store the converged atrophy factor as a state variable for post-processing, then calculate the atrophy part and the elastic part of the deformation gradient **F**^*a*^ and **F**^*e*^ (Eq. 4). We then calculate Cauchy stress, *σ* = *J*^−1^**PF**^*T*^, and its Jaumann rate,

(13) $${\text{c}}^{\text{abaqus }}=\text{c}+\frac{1}{2}[\sigma \bar{⊗}I+I\bar{⊗}\sigma +\sigma \underline{⊗}I+I\underline{⊗}\sigma ],$$

with the consistently linearized tangent stiffness matrix, c,

(14) $$c=\frac{1}{J^{e}}\left[I⊗F^{e}\right]:\frac{\partial^{2}\psi }{\partial F^{e}⊗\partial F^{e}}:\left[\text{I}⊗F^{eT}\right],$$

where we used the tensor operators ${\left\{•\bar{⊗}\circ \right\}}_{ijkl}={\left\{•\right\}}_{ik}⊗{\left\{\circ \right\}}_{jl}$ and ${\left\{•\underline{⊗}\circ \right\}}_{ijkl}={\left\{•\right\}}_{il}⊗{\left\{\circ \right\}}_{jk}$.

### 2.3 Finite Element Model Generation

**FIGURE 2 |:**
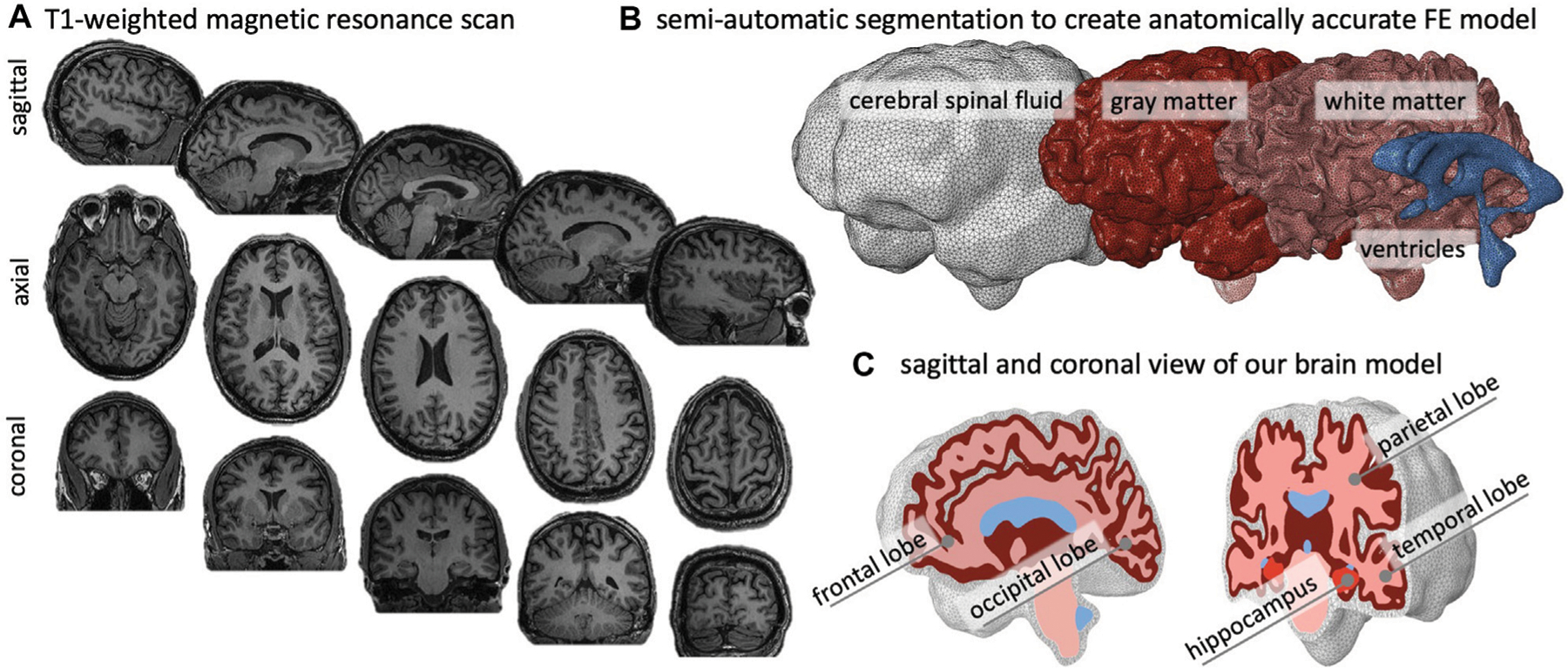
We create an anatomically accurate finite element model of the brain based on semi-automatic segmentation of a T1-weighted MRI. **(A)** The brain’s primary cortical and subcortical structures, as well as fluid volumes, are clearly visible in the representative sagittal, axial, and coronal slices shown here. **(B)** For the brain, we reconstruct the ventricles, white matter (WM), and gray matter (GM); we encase GM by cerebrospinal fluid (CSF) and approximate the skull by imposing zero-displacement boundary conditions on the CSF’s outer surface. **(C)** We create the GM layer by projecting the WM surface outward; this approach minimizes self-contact of the outer GM surface and provides an FE mesh that does not prevent sulcal widening due to shared nodes on the GM surface.

We created an anatomically accurate FE brain model from T1-weighted magnetic resonance images of a healthy adult male brain. We used ScanIp from Simpleware (Synopsis Inc., Mountain View CA) to semi-automatically segment the regions of interest and generate the FE mesh. Our model differentiates between gray matter (GM), white matter (WM), the hippocampus, ventricles, and cerebrospinal fluid (CSF). Figure 2A) shows representative sagittal, axial, and coronal MRI slices of the subject’s brain, as well as the volumetric reconstructions of the respective substructures. We built our model sequentially and began segmentation with reconstruction of the ventricles, followed by WM, GM, and finally CSF. We avoided reconstructing the skull by defining zero-displacement Dirichlet boundary conditions on the peripheral surface of CSF. Here, we merged the lateral ventricles, third ventricle, and fourth ventricle into a single volume in order to quantify ventricular enlargement, one of the hallmark features of brain aging. We paid close attention to the segmentation of WM tissue to accurately capture individual sulci and gyri across all lobes. To realistically simulate cortical thinning and sulcal widening, we must prevent self-contact of the cortical layer. Therefore, we inflated the WM segmentation by a constant thickness of 3 mm to obtain the GM layer. We then manually modified the GM layer to remove self-contact between lobes and folds in each slice. Ultimately, we aimed for a balance between agreement of segmentation and MRI on the one hand, and obtaining a FE mesh that may realistically predict structural shape changes of the brain on the other. Following WM and GM segmentation, we isolated the hippocampus as a separate substructure, given its relevance in AD as one of the first brain structures to markedly shrink. Finally, we inflated the GM layer by 5 mm and applied smoothing to obtain the CSF layer. This layer allows us to anchor the brain in our atrophy simulations while minimizing external forces on the GM layer.

#### Model Properties:

Our model consists of 1,361,277 tetrahedral elements: 7,925 elements for the ventricles, 2,898 elements for the hippocampus, 121,904 elements for WM, 172,238 elements for GM, and 98,755 elements for CSF. We restricted element edge length to vary from 2.0 to 2.3 mm to minimize element distortion and obtain similarly sized elements. We imported the mesh into Abaqus for analysis. Specifically, we use linear tetrahedral elements C3D4 and define two simulation cases. We simulate healthy aging by simply solving the atrophy problem and simulate accelerated aging by running a thermo-mechanical analysis. In both cases, we only prescribe zero-displacement Dirichlet boundary conditions to the outer surface of the CSF layer to fix the model in space. In the AD case, we additionally prescribe an initial concentration of *c*_0_ = 0.3 in the hippocampus. We used model parameters from our previous experimental and computational studies (Schaer et al., 2008; Weickenmeier et al., 2018; Weickenmeier et al., 2016) and summarize the model parameters for the atrophy and protein problem (Eq. 1, Eq. 5, Eq. 10) in Table 1. To assess long-term brain shape changes we simulate an age range of 40 years. Literature provides a myriad of large cohort studies that assess volumetric changes across this age-range (Apostolova et al., 2012; Coupé et al., 2019). Moreover, this allows us to review the impact of AD-onset time by varying the critical prion load necessary to trigger accelerated aging.

### 2.4 Data Analysis

We wrote custom python codes for post-processing of our simulations in order to determine volume ratios, anterior-posterior variations of the gyrification index, sulcal widening, and cortical thinning.

To calculate relative volume ratios of WM, GM, hippocampus, and ventricles, we sum the volume of all elements belonging to one of these subregions and divide by the total brain volume; we repeat this step for each time increment to obtain longitudinal changes as shown in Figure 7.

The gyrification index (GI) is determined by slicing our 3D model into 160 coronal slices (1 mm spacing between slices) and creating a binary image showing the domain associated with brain tissues, i.e., GM, WM, hippocampus, and ventricles wherever present. The subsequent steps are based on functions in the scikit-image processing package. Specifically, we determine the convex hull that fully encapsulates the brain domain to obtain the smoothed outer circumference and extract the contour tightly lining the pial surface. We repeat this process for each slice and determine the gyrification index as the local ratio between exact pial surface length and smooth outer circumference, as shown in Figure 11.

Our cortical thickness measurement is based on the approach used in FreeSurfer (http://surfer.nmr.mgh.harvard.edu) (Han et al., 2006). We create triangulated surfaces of the outer GM surface and the outer WM surface and define cortical thickness *t*_*c*_ as the average of two distance measures, *d*_*ij*_ and *d*_*jk*_. We iterate over every node of the GM surface, *n*_*i*_, identify the closest node on the WM surface, *n*_*j*_, and save the Euclidian distance between these two nodes as *d*_*ij*_. We repeat this search for that particular WM node, *n*_*j*_, and save the Euclidian distance between *n*_*j*_ and GM node *n*_*k*_ as distance *d*_*jk*_. We ultimately obtain a cortical thickness measure at each GM surface node as *t*_*c*_ = 0.5[*d*_*ij*_ + *d*_*jk*_] and plot the result as a surface plot, as shown in Figure 8. We export nodal coordinates of our surfaces in the undeformed and the deformed configuration in order to determine cortical thickness at a young and an old age.

We introduce sulcal widening as the volume increase in the fluid-filled cavity of five prominent sulci, i.e., the intra-parietal sulcus, the superior temporal sulcus, the central sulcus, the sylvian fissure, and the superior frontal sulcus, as shown in Figure 10. Similar to determining the relative volume fractions, we sum the volume of all elements of a particular sulcal fold for each time increment of our simulation.

## 3 RESULTS

We evaluate our simulations with respect to hallmark features of cerebral atrophy and aim at identifying key differences between healthy brain aging and accelerated aging associated with AD.

### 3.1 Spatiotemporal Progression of Toxic Proteins in Alzheimer’s Disease

**FIGURE 3 |:**
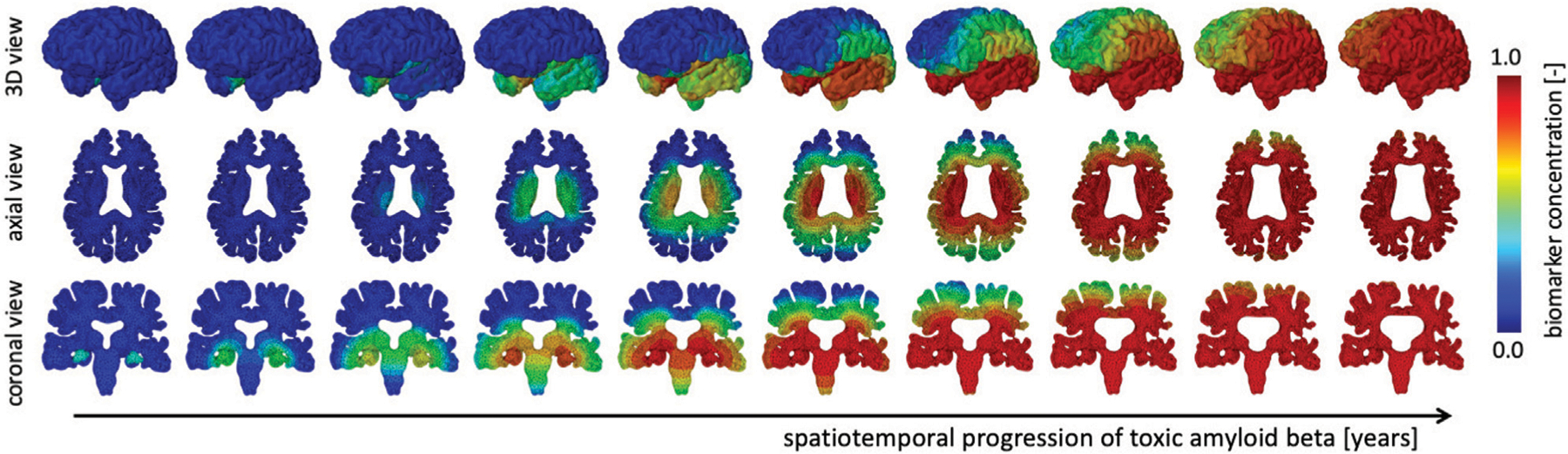
The spatiotemporal spreading behavior of our biomarker for neurodegenerative disease is governed by a reaction-diffusion equation. We seed the biomarker in the hippocampus and observe a gradual infiltration of the whole brain. The temporal lobe is affected first, followed by the occipital, then parietal, and finally the frontal lobes, see 3D view. Moreover, we observe an early affect on deep gray and white matter structures before diffusing outward into the cortical layer, see axial view. In our current version of the model, we prescribe equal diffusion in gray and white matter tissue, which is reflected in the diffuse spreading of the biomarker concentration.

We simulate the spreading of neurofibrillary tangles (NFT) consisting of misfolded tau protein based on the toxic protein spreading model described in §2.1. Pathological studies have shown that NFTs first appear in the entorhinal cortex and subsequently spread throughout the brain. Figure 3 shows the spatiotemporal propagation of the NFT concentration through the brain. We observe that the hippocampus is affected first, then infiltrates the temporal lobe next, followed by the parietal lobe, occipital lobe, and in the late stages reaches the frontal lobe. Our observations are in line with cadaver studies that show a similar progression pattern of NFTs (Jucker and Walker, 2018). The coronal view shows a highly symmetric protein spread in the left and right hemisphere; from the axial and coronal cross-sections, it can be seen that deep gray matter structures tend to saturate with NFTs first. Early deep gray matter involvement, such as putamen and thalamus (de Jong et al., 2008), is linked to well-known early symptoms of AD, including short-term memory loss, difficulty performing daily tasks, and mood changes. The delay between onset and cortical layer involvement is part of the long pre-symptomatic phase of AD (Hanseeuw et al., 2019) and consistent with imaging studies that observed spatially heterogeneous atrophy patterns (Anderson et al., 2012).

### 3.2 Spatiotemporal Distribution of the Atrophy Factor in Healthy Brain Aging and Alzheimer’s Disease

**FIGURE 4 |:**
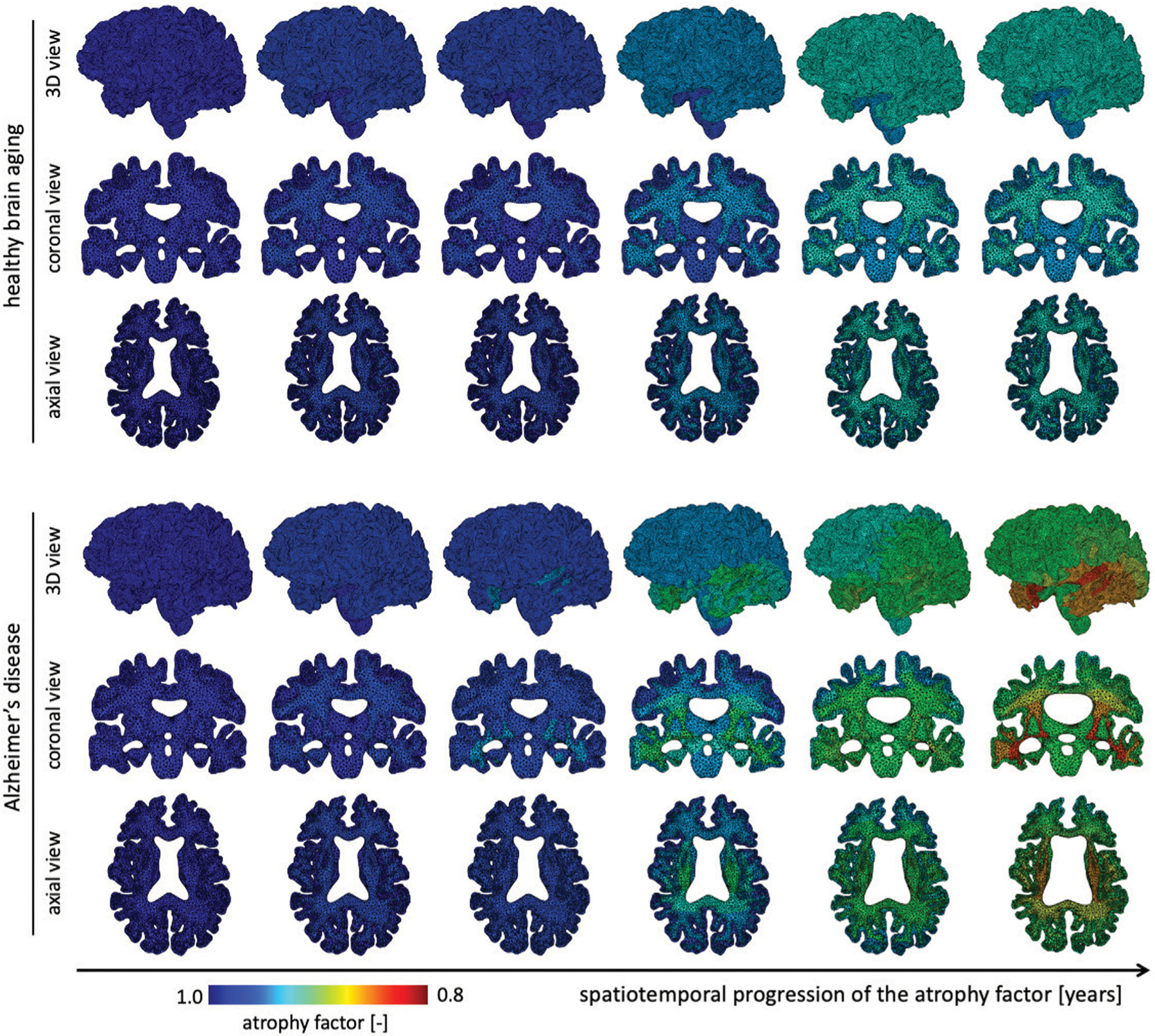
We show the spatial distribution of the atrophy factor over our simulation period of 40 years. In our model we differentiate between healthy (top rows) and accelerated, or pathological, aging (bottom rows). We prescribe a constant, albeit different, atrophy rate for gray and white matter tissue in healthy aging. In pathological aging, the atrophy factor in Alzheimer’s disease is coupled to the biomarker concentration and increases the atrophy factor once biomarker concentration exceeds a critical value; therefore, the AD-related atrophy factor follows a similar spatiotemporal progression pattern as the biomarker concentration. Atrophy factor of one corresponds to no volume change and we observe a maximum volume loss of 0.798. Since cross-sectional studies have identified more white matter volume loss in comparison to gray matter, we prescribe a higher atrophy rate which leads to more pronounced WM atrophy, see coronal and axial view.

The atrophy model allows us to differentiate between healthy and AD aging. On top of an age-proportional atrophy factor in healthy aging, we added additional toxic protein concentration-related atrophy to simulate AD. Figure 4 shows the spatiotemporal distribution of the atrophy factor, i.e. the volume shrinking fraction, which ranges from 1 (no shrinking) to 0.8 (maximum volume loss). We differentiate between WM and GM atrophy rates due to tissue specific neurodegenerative processes. Therefore, GM and WM have the same atrophy factors in healthy aging, respectively. In AD, we see a spatially heterogeneous distribution with maximum atrophy in deep WM and GM structures and in the frontal lobe. The coronal view shows that the cortex exhibits an atrophy gradient that ranges from the temporal lobe to the frontal lobe; in WM we observe a gradient ranging from the temporal lobe to the parietal lobe. Both are consistent with imaging studies investigating regional atrophy rates in the cortex (McDonald et al., 2009).

### 3.3 Brain Deformations in Healthy Brain Aging and Alzheimer’s Disease

**FIGURE 5 |:**
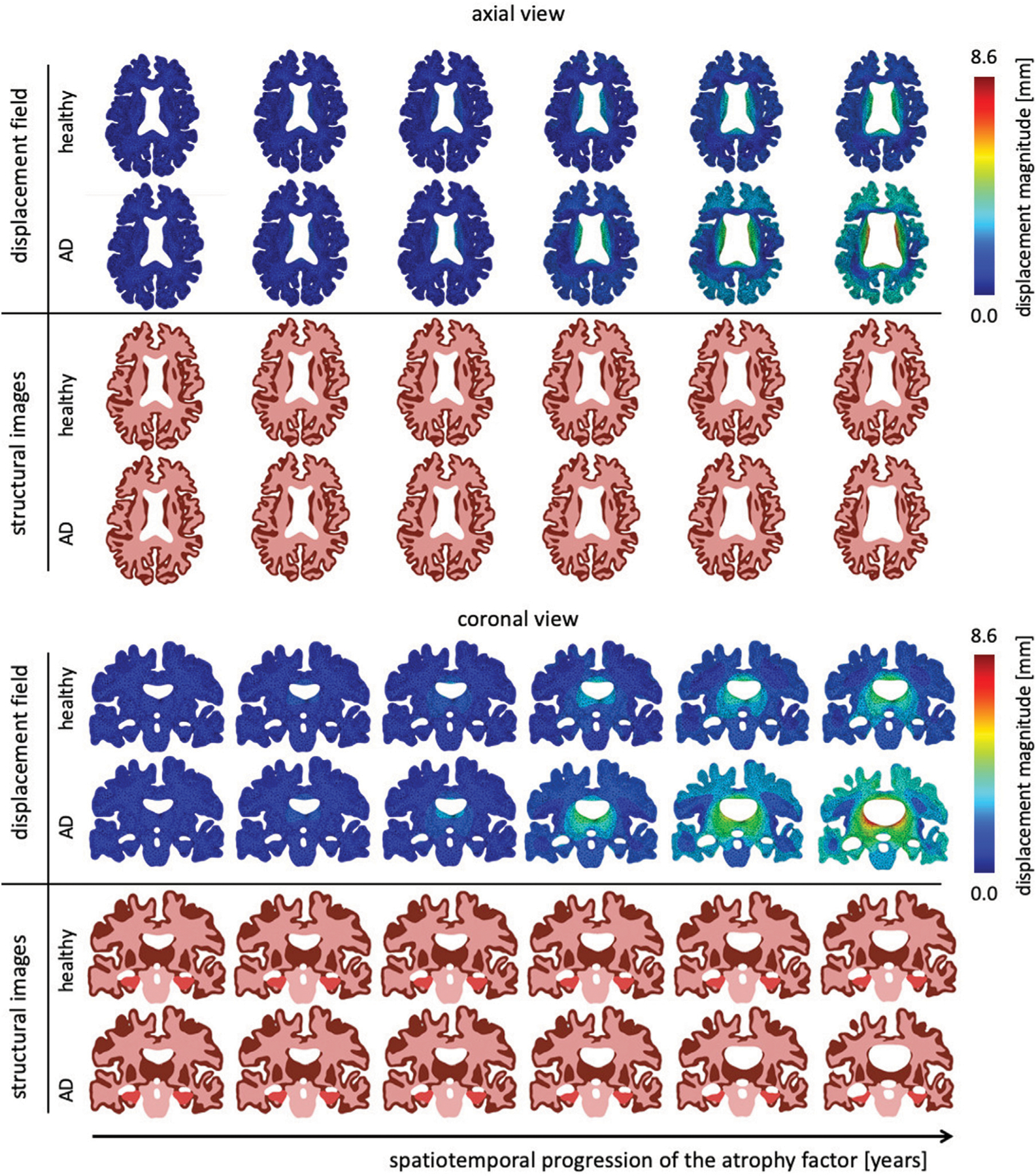
Representative axial and coronal views of the displacement magnitude and structural images at six time points during the aging process. We show healthy aging and Alzheimer’s disease-related aging in the top and bottom rows, respectively. Brain deformation is higher in Alzheimer’s disease than healthy aging, and is largest around the ventricles. Moreover, we observe significant enlargement of the ventricular horns in the vicinity of the hippocampus, see coronal view. The forth time point clearly shows a distinct separation of the displacement trajectories.

Figure 5 shows the temporal progression of the predicted deformation field and corresponding equivalent structural image for healthy aging and AD for representative axial and coronal sections. We observe maximum displacement magnitudes of 7.17 mm for healthy aging and 8.58 mm in AD. Maximum displacements concentrate around the lateral ventricles which undergo significant enlargement, especially in the AD brain. In comparison to the atrophy factor, which affects the hippocampus first, ventricles, and the surrounding white and gray matter regions, appear to deform early, followed by cortical deformations. For late stages we observe higher displacement magnitudes for the GM layer in comparison to deep white matter structures. The structural scans reveal hallmark features of cerebral atrophy: hippocampal shrinking, early onset of deep GM shrinking, cortical thinning, and ventricular enlargement. We generally observe that these features are exacerbated in AD in comparison to healthy aging. These observations are strongly correlated with medical imaging based studies that observe hippocampal shrinking, cortical thinning, and ventricular enlargement as early predictors for AD (Apostolova et al., 2012). Previous computational studies typically prescribe a zero-boundary condition on nodes of the brainstem in order to fix the model in space (Harris et al., 2019; Schäfer et al., 2019). These boundary conditions significantly impact the simulated deformation field and limit these models’ abilities to resolve temporospatial patterns or critical features such as ventricular enlargement. Here, the cerebrum is loosely tethered to the skull *via* the ultrasoft CSF layer which allows for physical features to emerge naturally. Strikingly, we observe global brain involvement despite scattered atrophy features.

### 3.4 Ventricular Enlargement in Healthy Brain Aging and Alzheimer’s Disease

**FIGURE 6 |:**
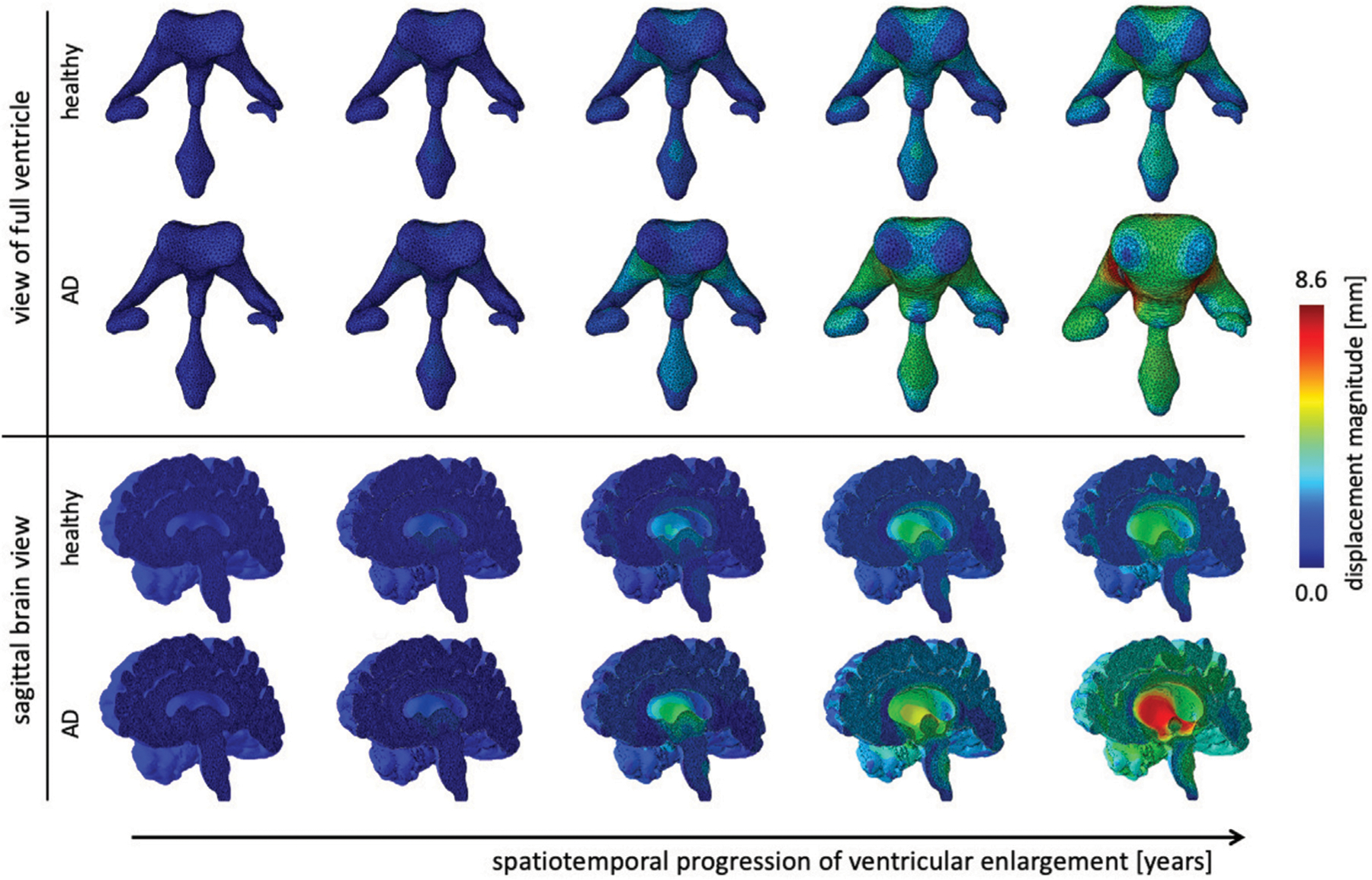
Ventricular enlargement is one of the most prominent features of the aging brain. The ventricular body expands most and the anterior and posterior horns inflate in response to tissue loss. Alzheimer’s disease has a larger affect than healthy aging. Overall the ventricular volume more than doubles in Alzheimer’s disease and increases by 165% in healthy aging. The sagittal view of the brain shows the effect on deep gray matter structures.

Figure 6 shows the gradual expansion of the lateral ventricles for healthy aging and AD. We observe significantly larger ventricles in AD, which increase by a factor 2.66, in comparison to healthy aging, where ventricles increase by a factor 1.76. The simulation predicts a predominantly uniform inflation of the entire ventricular cavity in healthy brain aging at a moderate expansion rate. In AD, we observe consistent overall ventricular dilation, but notice a significant concentration of maximum expansion in the body of the ventricles and the posterior horns. This observation is consistent with a medical imaging study that reported a temporal pattern that starts in the occipital horn, then affects the body, and ultimately reaches the frontal horns (Apostolova et al., 2012). The sagittal view of the brain shows the corresponding white and gray matter loss. As the ventricles expand, we observe a smoothing of the superior horn, temporal horn, and occipital horns with an overall decrease in curvature of the ventricular surface.

## 4 DISCUSSION

### 4.1 The Origin of Brain Volume Loss

Cerebral atrophy is caused by diverse tissue damage mechanisms that culminate in brain volume loss (Oschwald et al., 2020; Blinkouskaya et al.). While healthy aging and AD share some of the gray and white matter damage mechanisms there is a distinct point during the lifespan where the atrophy trajectory in AD diverges from the healthy model due to accelerated neurodegeneration (Callaghan et al., 2014; Coupé et al., 2019). Most common damage mechanisms are neurodegeneration in GM (Farokhian et al., 2017), demyelination in WM (Vernooij et al., 2008), activation of microglia cells (Von Bernhardi et al., 2015), and cerebral small vessel disease which is associated with microbleads, lacunes, and perivascular spaces (Cuadrado-Godia et al., 2018).

In gray matter, neurons undergo morphological changes linked to a reduction in the complexity of dendrite arborization (Dickstein et al., 2007). The underlying dendritic shortening and loss of dendritic spines leads to a progressive decrease in synaptic density and synaptic transmission with major implications on cognitive decline (Dickstein et al., 2013). Unlike healthy aging, AD is accompanied by neuron death due to the ever-increasing presence of neurotoxic proteins such as amyloid beta plaques and neurofibrillary tangles (Serrano-Pozo et al., 2011). GM volume loss is therefore exacerbated in AD and manifests in accelerated atrophy rates (Anderson et al., 2012) and increased cortical thinning (Du et al., 2007). It is well established today that the very first morphological changes associated with AD appear in the entorhinal cortex and hippocampus at least 10 years before the diagnosis (Dickstein et al., 2007).

In WM the most prevalent tissue changes are characterized by partial loss of myelin, axons, and oligodendroglial cells (Xiong and Mok, 2011); mild reactive astrocytic gliosis linked to WM lesions (Rodríguez-Arellano et al., 2016); arteriolosclerosis of small vessels resulting in incomplete ischemia and cell death (Pantoni, 2002); and the emergence of perivascular spaces that interfere with the glymphatic drainage of the brain’s waste products (Rasmussen et al., 2018; Wardlaw et al., 2020).

During normal aging, amyloid beta plaques can be found in the frontal lobe, hippocampus, and entorhinal cortex of healthy elderly. In addition, neurofibrillary tangles, although much rarer than plaques, are commonly found in the medial temporal areas after 50 years of age (Dickstein et al., 2007). In AD, however, the progressive aggregation of plaques and NFTs has detrimental effects on neuronal morphology and synapses. Unlike in normal aging when neurons shrink, AD triggers sustained neuronal loss in neocortical and entorhinal regions of up to about 30% (Mattson, 2004).

### 4.2 Atrophy Dynamics During Aging

**FIGURE 7 |:**
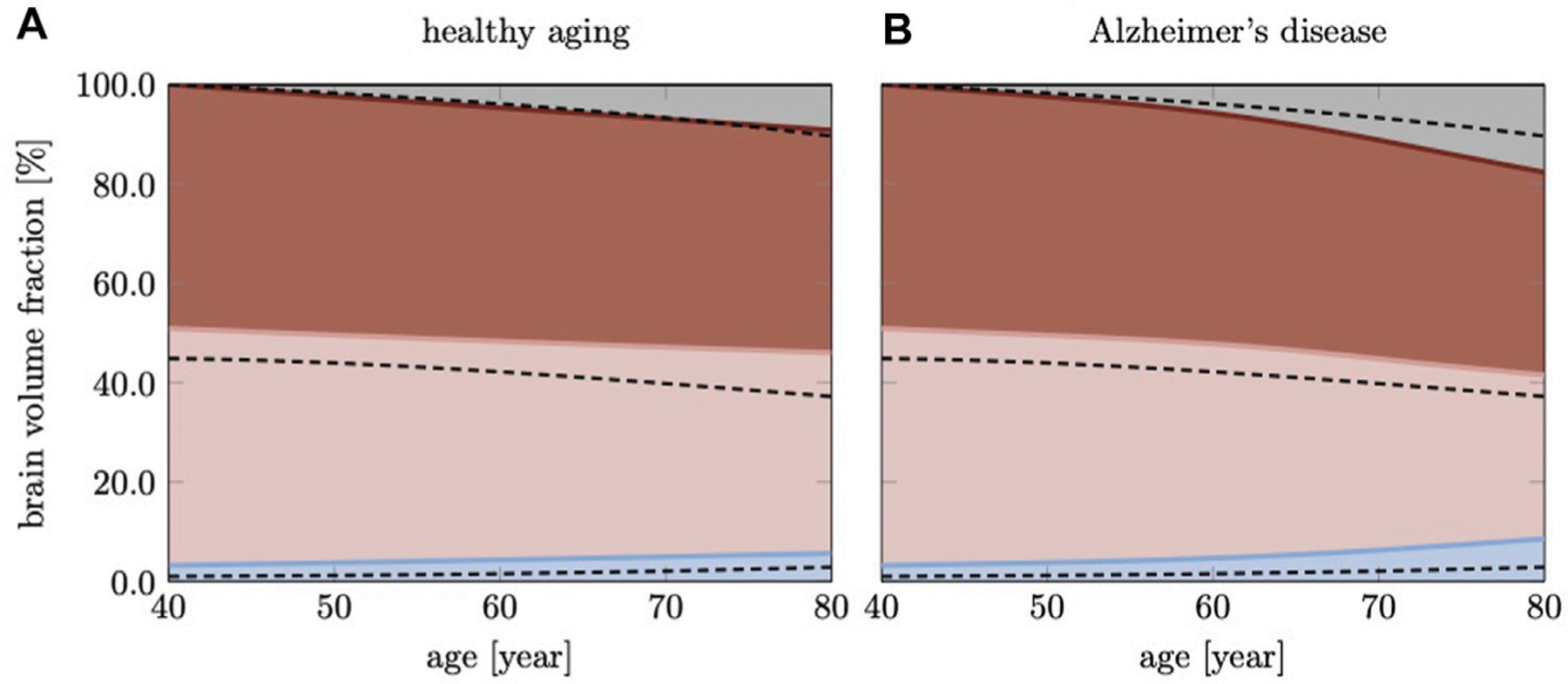
Cross-sectional studies, with subjects covering many decades of life, provide insight into the transient brain volume changes and how they break down into the brain’s cortical and subcortical regions. Here, we compare our model’s predicted gray matter, white matter, and ventricular volume fraction with data reported by Coupe et al. (2019) for **(A)** healthy aging and **(B)** AD. In AD, we clearly observe a deviation from healthy aging in the form of accelerated atrophy. The grey area shows the loss of tissue volume that is replaced by fluid.

Figure 7 shows brain volume fractions of GM, WM, and ventricles representative of a brain aged 40 years and older. We extracted atrophy data from Coupe et al. who identified volume changes from a cross-sectional study with 4,329 subjects (2,944 healthy subjects and 3,262 subjects with AD and mild cognitive impairment) (Coupé et al., 2019), see dashed lines. We focus on brain aging and calibrate our model parameters such that our model provides good qualitative agreement for healthy brain aging, (Figure 7A). Our model successfully reproduces GM and WM volume loss and ventricular enlargement. The offset between GM, WM, and ventricular volume fractions is due to comparison of a personalized brain model with cross-sectional data. More importantly, the numerically observed atrophy trajectories paint a representative picture that demonstrates the ability of our modeling approach to predict shape changes associated with brain aging. Our model predicts GM volume fraction to drop from 52.36% at age 40 years to 50.49% at age 80 years in healthy aging and 49.34% in AD; WM volume fraction to drop from 47.63% at age 40 years to 40.29% at age 80 years in healthy aging and 32.95% in AD; ventricular volume fraction increases from 3.22% at age 40 years to 5.66% at age 80 years in healthy aging and 8.57% in AD. AD clearly exacerbates tissue loss and exhibits an accelerating atrophy rate with increasing age, (Figure 7B). Tissue lost due to atrophy is replaced by fluid (volume fraction shown in grey) linked in one part to ventricular enlargement and in another part to sulcal widening and loss of gyrification (Scahill et al., 2003).

### 4.3 Cortical Thinning

**FIGURE 8 |:**
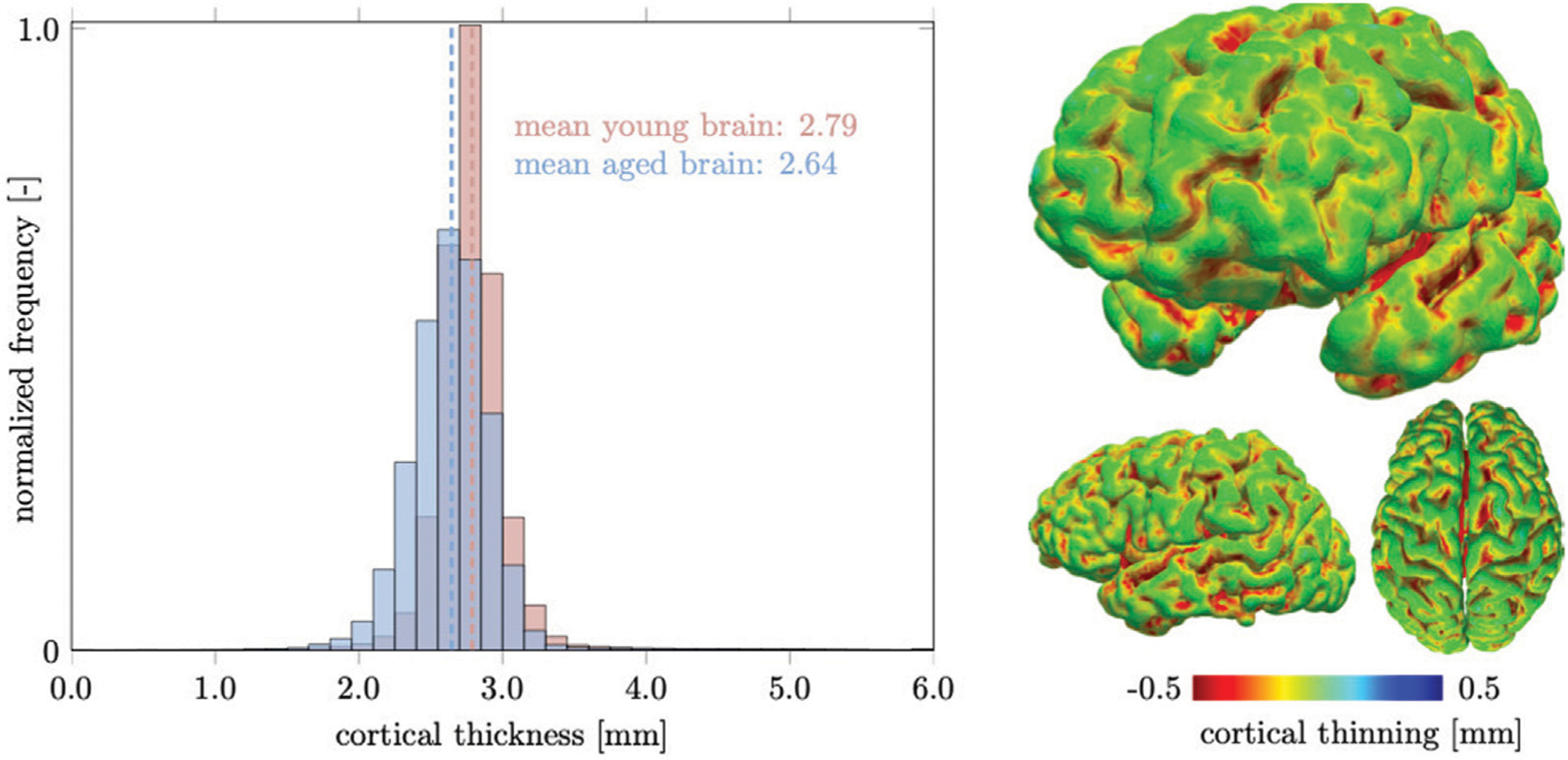
We measure cortical thickness changes in the healthily aging brain and observe a clear difference between increased sulcal thinning in comparison to gyri that remain nearly unchanged. Only few locations are predicted to thicken and are located in deep gray matter locations. Overall the mean cortical thickness decreases from 2.79 mm in the young brain to 2.64 mm in the aged brain.

The cortical layer is subject to spatially heterogeneous age-related cortical thinning. The deterioration of dendritic connections and the loss of GM neurons cause volume loss that can be broken down into cortical thickness and surface area. These two properties do not necessarily follow each other chronologically (Dickerson et al., 2009). The differentiation between both measures has proven useful, however, because of increased sensitivity with respect to age-related changes (Storsve et al., 2014; Dotson et al., 2016). In our computer model, we observe a mean cortical thickness of 2.79 mm in the young brain and 2.64 mm in the aged brain. In Figure 8, we report our model’s brain thickness which ranges from 1.5 to 4.3 mm in the young brain and decreases to a range from 1.3 to 3.9 mm in the aged brain. These values compare well to results presented by Fjell et al. who observed a progressive decline in overall cortical thickness in their three subject groups aged < 40, 40–60, and > 60 (Fjell et al., 2001; Fjell and Walhovd, 2010). They report that sulci undergo more pronounced thinning than gyri and that thinning is unevenly distributed across the cortex. Based on data extracted from Fjell et al., the cortex appears to thin by roughly 0.1% per year, or 0.00745 mm, which corresponds to an overall thickness decrease of about 0.3 mm over the course of 4 decades for subjects aged > 40 (Fjell et al., 2015). The linearly decreasing relationship between cortical thickness and age across several datasets provides strong support for our modeling approach which assumes a constant atrophy rate for all ages (Fjell et al., 2001; Du et al., 2006). Despite significant efforts to identify common thinning trajectories in the human brain, cortical thinning is driven by molecular and cellular processes that are not limited to individual regions. Cross-sectional studies report that the frontal cortices are most strongly affected and that the medial-temporal cortices, i.e., parahippocampal and entorhinal cortex, are moderately affected. Lateral inferior parts of the temporal lobes show least thinning and the superior parts of the lateral temporal lobes exhibits more pronounced thinning than the inferior parts (Fjell et al., 2001; Fjell and Walhovd, 2010). In our model, we observe slightly higher thinning in the frontal and temporal region, while the occipital lobe thins less. In aging research the temporal lobes play a significant role because they are functionally related to the hippocampus and other GM structures that are associated with memory loss and cognitive decline (Dickerson et al., 2009; Dhikav et al., 2014). In the end, our model leads to fairly similar cortical thinning across the entire brain due to the prescribed constant GM atrophy rate. Coupling to the spreading of neurotoxic proteins may lead to a stronger heterogeneity in terms of thinning.

### 4.4 Hippocampal Shrinking and Ventricular Enlargement

**FIGURE 9 |:**
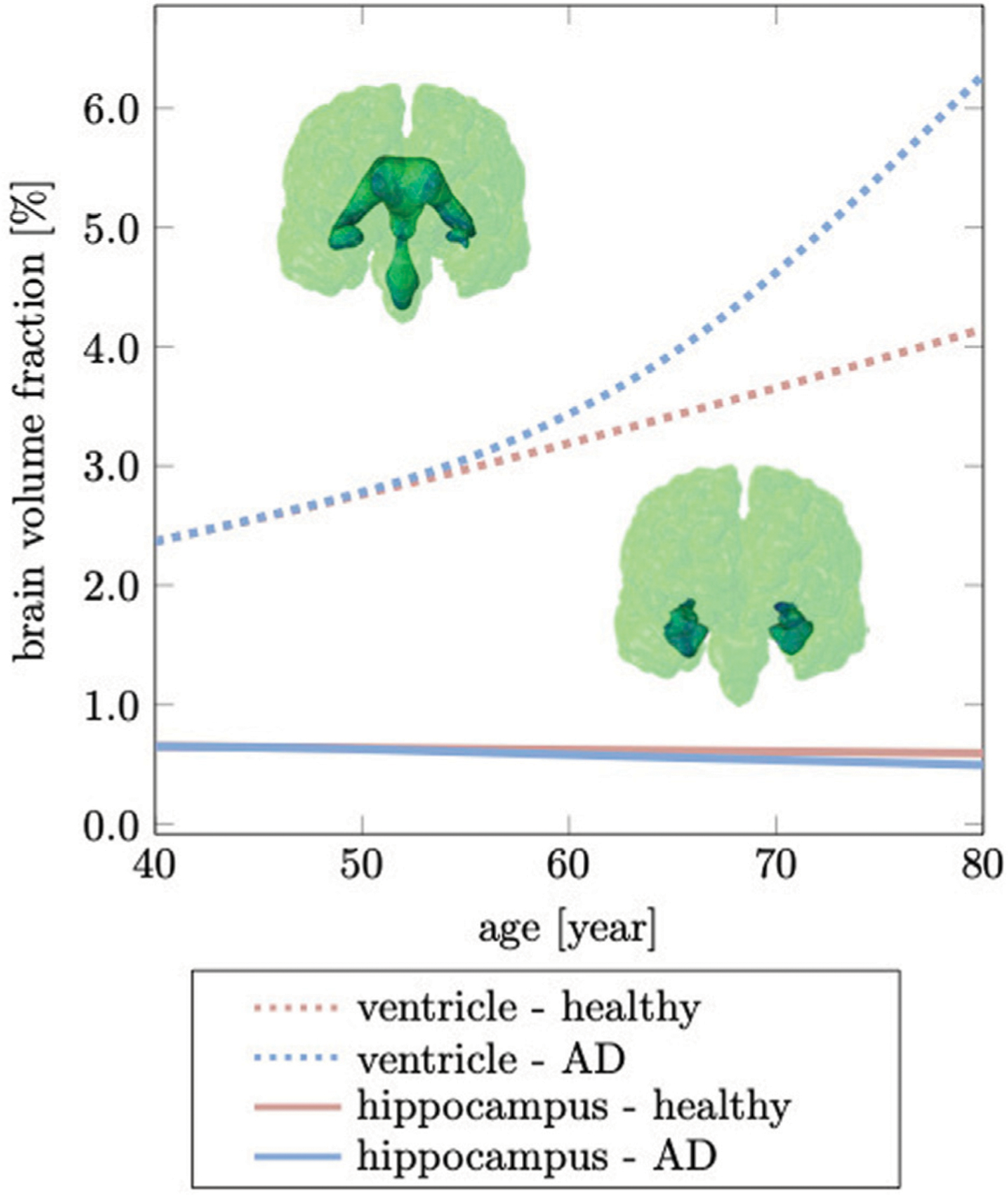
Hippocampal shrinking and ventricular enlargement differ for healthy aging and Alzheimer’s disease. The initial overlap between healthy aging and Alzheimer’s disease is due to the gradual spread of our biomarker through the brain which ultimately accelerates brain changes passed the age of 60 years. This deviation from the healthy trajectory is used as a biomarker for detecting Alzheimer’s disease (Apostolova et al., 2012).

The hippocampus is one of the, if not, the earliest cortical substructures to undergo detectable atrophy in Alzheimer’s disease and related dementias (Henneman et al., 2009). Hippocampal changes can be detected as early as 10 years prior to the onset of symptoms and is therefore considered to be a strong indicator for abnormal aging processes (Ritchie et al., 2016; Kinnunen et al., 2018). Hippocampal shrinking precedes most cortical changes by up to 5 years and is reported to shrink by 5.2% per year based on data from cross-sectional brain imaging studies (Thompson et al., 2004; Henneman et al., 2009). It is primarily linked to de-arborization of subcortical GM neurons (Esiri, 2007; Dickstein et al., 2013). In comparison to healthy aging, Alzheimer’s disease accelerates neuronal degeneration due to accumulation of neurotoxic amyloid beta plaques and neurofibrillary tangles (Bobinski et al., 1999). Figure 9 shows our model’s predicted volumetric shrinking for healthy aging and AD. We observe a decrease of the hippocampal brain volume fraction by 8.87% for healthy aging and by 24.1% for AD. The direct comparison illustrates the distinct difference in the atrophy trajectory in accelerated aging in AD observed in cross-sectional studies (Coupé et al., 2019).

The brain tissue volume lost due to cerebral atrophy, is replaced by fluid. Structurally, this manifests in significant ventricular enlargement (Pagani et al., 2008; Apostolova et al., 2012) and an increase in the space between folds, i.e., sulcal widening (Liu et al., 2013; Jin et al., 2018). Ventricular enlargement is one of the most prominent features in longitudinal medical images and represents a major change in brain topology (Sengoku, 2020). Mechanically, the extent of ventricular enlargement is significant and will lead to high loads on the membrane separating ventricle and cerebrum. The ependymal cells lining the ventricular wall are likely to be fatigued with age, leading to CSF leakage into white matter and causing tissue degeneration, such as leukoaraiosis in the vicinity of ventricular horns (Milhorat et al., 1970; Todd et al., 2018). Our model predicts a uniform volumetric expansion of the entire ventricles which is reflective of findings from imaging studies (Salat et al., 2009; Coupé et al., 2019). Our simulation is able to reproduce this deformation mode due to our physically motivated boundary conditions on the FE model. Instead of constraining individual nodes in the brainstem (Harris et al., 2019; Schäfer et al., 2019), here, we *suspend* the brain inside the skull by mimicking CSF as an ultrasoft, highly compressible solid. The suspension of the shrinking cerebrum allows for the ventricles to expand. This leads to a fairly symmetric displacement field with respect to the left and right hemisphere. In our model, the initial ventricular volume corresponds to 2.37% of the total intracranial volume. In our simulation, we observe an increase to 4.15% of total intracranial volume, or a 75.03% volume increase in healthy aging; In AD, ventricular volume fraction increases to 6.28%, or an overall volume increase by 164.98%. Our data aligns well with data reported by Coupe et al. that observe significant acceleration of ventricular expansion at age 40 (Coupé et al., 2019). Microstructurally, ventricular expansion is accompanied by a progressive deterioration of the ventricular wall which is composed of ciliated ependymal cells that undergo significant cellular stretch during each pulsation cycle. Over the course of a lifetime, these cells accumulate significant mechanical fatigue and cause membrane failure (Milhorat et al., 1970; Jiménez et al., 2014). The subsequent leakage of CSF into white matter tissue causes leukoaraiosis and white matter deterioration.

### 4.5 Sulcal Widening and Loss of Gyrification

**FIGURE 10 |:**
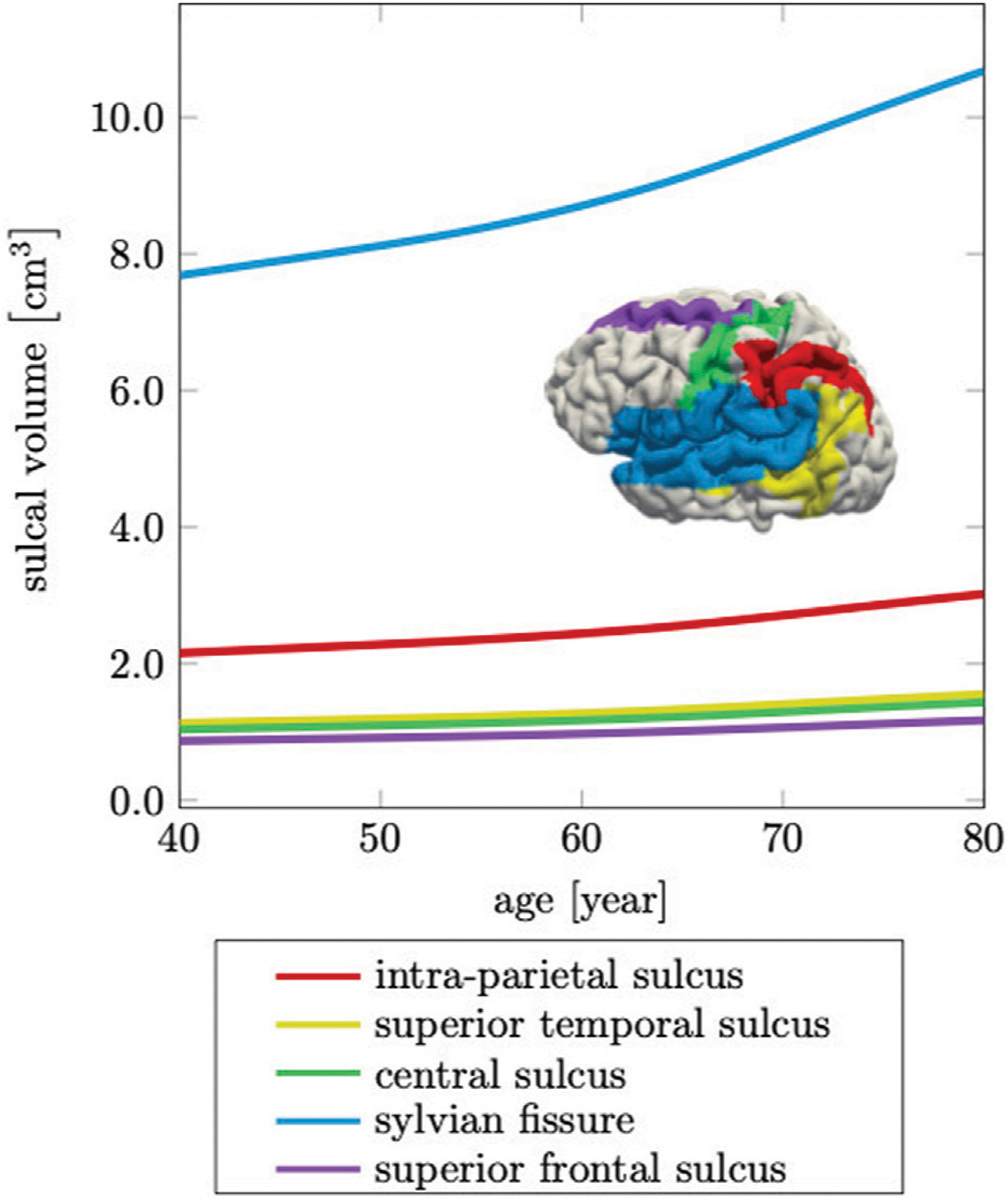
Cerebral atrophy includes sulcal widening, or the increase in intra-sulcal volume due to the shrinking of surrounding tissue. The sylvian fissure, which separates the frontal and parietal lobes from the temporal lobe, increases most by 39%, while the other sulci increase on average by 36% over a 40 years time period.

Ventricular enlargement is accompanied by an increase in the space between folds and loss of gyrification (Hamelin et al., 2015; Aso et al., 2020). This feature is less prominent on medical images, but is another indicator for the significant topological changes of the brain (Plocharski et al., 2016; Shen et al., 2018). From a FE modeling perspective, creating an anatomically accurate mesh that properly capture sulcal widening represents a major challenge. Most folds touch each other such that the segmentation process typically does not produce a GM surface without self-contact. This leads to node sharing of elements that belong to different folds and ultimately, prevents models to allow for separation of the GM surface upon tissue atrophy. Here, we specifically address this issue and produced a FE mesh that has minimal node sharing between neighboring folds. Therefore, our model exhibits this hallmark feature of cerebral atrophy and allows us to compare model response with imaging data. Jin et al., for example, report that the mean sulcal width between primary sulci increases by ~ 17.3% from 1.27 ± 0.17 mm in middle-aged persons to 1.49 ± 0.20 mm in older adults (71). In Figure 10 we report sulcal widening, a measure of the volume increase of the fluid between folds. We segment these volumes for five prominent sulci, the intra-parietal sulcus, the superior temporal sulcus, the central sulcus, the sylvian fissure, and the superior frontal sulcus (Kochunov et al., 2005; Liu et al., 2013). We observe that the overall volume change of all sulci follow a similar trend and increase by up to 40%. Similar to previous work, the sylvian fissure exhibits the largest increase in width and is noticeably larger in individuals with AD in comparison to cognitively normal subjects (Park et al., 2013; Cai et al., 2017). Overall, we observed that the technical challenges associated with detailed geometric interpretation of sulcal changes, such as sulcal widening and changes in sulcal depth, represent a barrier to serving as a reliable biomarker for morphological changes in the aging brain. Especially, subject-specificity will limit absolute comparisons with any healthy or diseased cohort (Shen et al., 2018).

**FIGURE 11 |:**
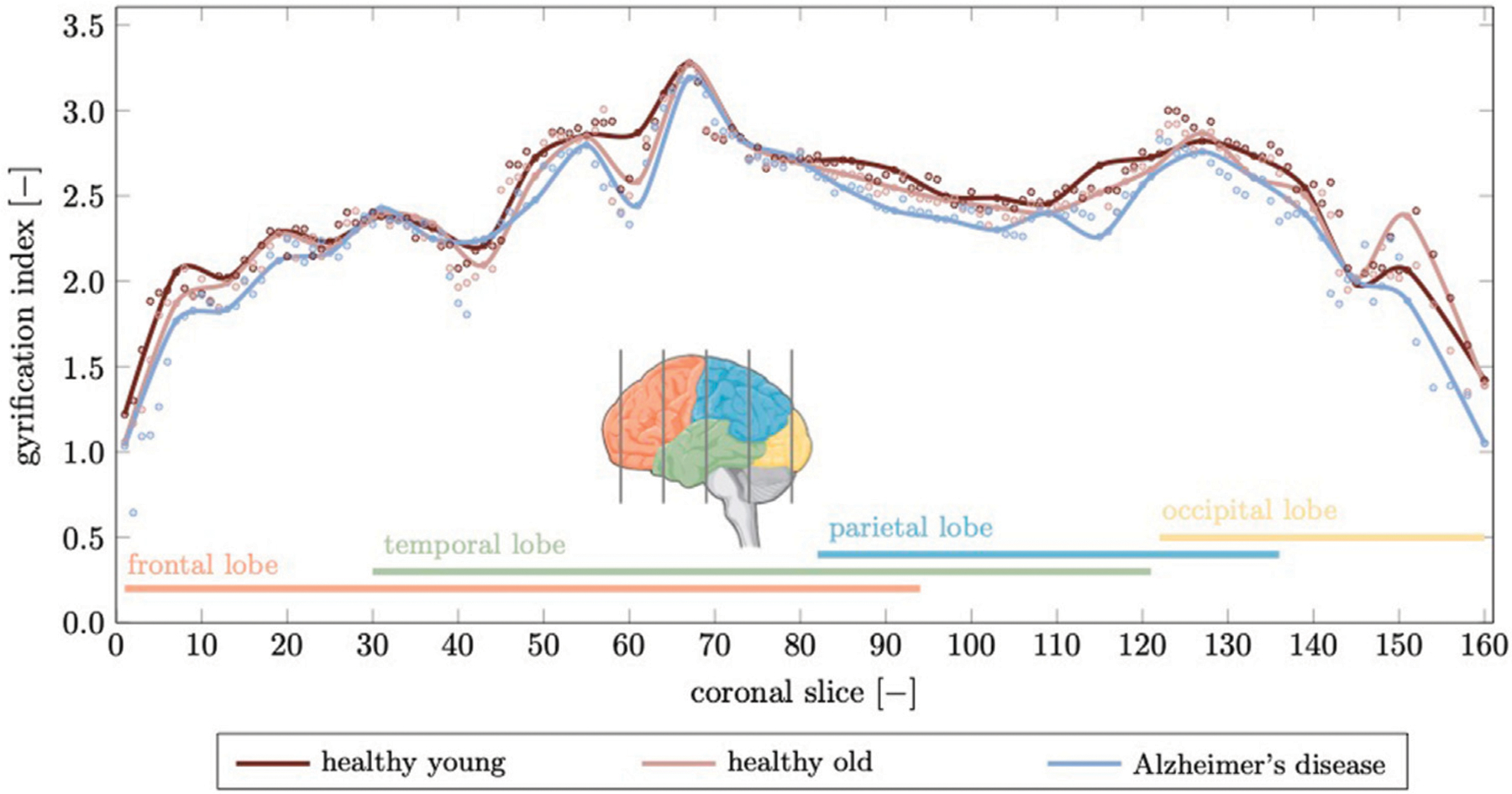
The gyrification index is a measure for the degree of folding. Here, we compute a gyrification index for 164 coronal slices generated from our finite element model. We compare the gyrification index for the young brain, aged brain, and in Alzheimer’s disease and observe a noticeable decrease in Alzheimer’s disease in the temporal and parietal lobes while the frontal lobe, which is affected least in terms of atrophy, shows only small deviations. Peak gyrification is observed in the vicinity of the sylvian fissure which is widens significantly as discussed before.

The gyrification index (GI), defined as the ratio between actual GM surface divided by the smooth surface surrounding the cortex, is another parameter that is closely linked to the topology of brain folds (Madan, 2021). In Figure 11 we show the gyrification index for 164 coronal slices calculated for the healthy young brain, healthy aged brain, and the brain affected by Alzheimer’s disease. The GI is highest across the brain for the young brain. With aging or AD, the GI decreases due to decreased folding. We observe the highest GI in the temporal lobe with 3.28 for young, 3.27 for aged, and 3.19 for the AD brain; minimum GI is observed in the frontal lobe with 1.22 in young, 1.06 in aged, and 0.64 in the AD brain. We observe a mean GI of 2.48 ± 0.38 in the young, 2.42 ± 0.4 in the aged, and 2.32 ± 0.44 in the AD brain. The most prominent and persistent drop in GI is observed in the temporal and parietal lobes which are heavily affected by early infiltration of our neurotoxic biomarker and corresponding accelerated atrophy. Our reported values compare well with cross-sectional studies reported in literature (Jockwitz et al., 2017; Madan, 2021). In a cross-sectional study by Cao et al., the GI drops from 3.4 at age 10 to 2.6 at age 85, following the curve GI = *a* + *b* ln (*A* + *c*), with age *A* and parameters *a* = 3.4, *b* −0.175, and *c* = −2.9991 (Cao et al., 2017). According to this formula, GI drops from 2.8 at age 40 to 2.6 at age 80, or by 4.5% between ages 40 and 80. Our model predicts a 2.7% change for the most folded coronal slice.

### 4.6 Limitations

Our computational model is based on several assumptions and thus not without limitations. For example, when creating the FE mesh, we uniformly inflate the WM surface to create a GM layer which results in a fairly homogeneous GM thickness across the brain. In reality, the gray matter layer is characterized by thickness differences between sulci and gyri (Lin et al., 2021) and varies across the brain (Fischl and Dale, 2000). We chose this approach due to the necessity to avoid self-contact between GM folds in order to capture sulcal widening during atrophy. Furthermore, our current constitutive model differentiates between GM and WM atrophy rates, but assumes a uniform parameter across the brain. Cross-sectional studies have demonstrated significant regional variation in brain shrinking rates in healthy aging and AD (Fox and Schott, 2004; Fjell et al., 2014). The coupling of biomarker concentration and atrophy rate in our model introduces, however, a degree of heterogeneity that exacerbates spatiotemporal differences between healthy aging and AD. Our model shows good agreement with cross-sectionally observed image-based atrophy patterns. Going forward, there is a need to develop a validation approach that allows to calibrate model parameters against longitudinal imaging data of individual subjects (Rusinek et al., 2003). To that end, we will develop a non-rigid registration technique that delivers the full-field displacements of the brain between two images (Wang et al., 2021). And lastly, AD is characterized by two different protein spreading mechanisms: connectivity-based spread *via* intracellular diffusion of neurofibrillary tangles along the axon network and proximity-based spread of amyloid beta *via* extracellular aggregation of plaques (Jack and Holtzman, 2013). Here, we only consider isotropic diffusion through the bulk tissue. As a next step, we will integrate the diffusion tensor imaging-based tractome to more accurately represent intracellular spreading of tau which has shown to better correlate with neurocognitive decline (Raj et al., 2015).

## 5 CONCLUSION

Brain shape undergoes many changes throughout life. Advanced aging is characterized by progressive atrophy which appears as brain volume loss, cortical thinning, sulcal widening, and ventricular enlargement. These morphological changes are part of healthy brain aging and it remains unclear how these changes relate to cognitive decline. In case of accelerated aging, such as in neurodegenerative diseases like AD, these structural changes are exacerbated due to the presence of neurotoxic proteins that spread through the brain. Here, we developed a constitutive framework for the simulation of three-dimensional morphological changes of the brain in healthy aging and AD. Our anatomically accurate FE model nicely captures volume loss, GM thinning, ventricular enlargement, and loss of gyrification. We compare our numerical results to commonly studied structural properties extracted from medical images and demonstrate that our generalized model shows good agreement with cross-sectional aging data. As a next step, we will utilize our modeling approach to create subject-specific FE models and validate our simulations against their longitudinal imaging data. This work has the potential to systematically investigate the impact of gray and white matter aging mechanisms, such as cerebral small vessel disease, leukoaraiosis, lacunes, and the dearborization of neurons, on the evolving morphology of the healthily and pathologically aging brain.

## Figures and Tables

**TABLE 1 |:** Multiphysics atrophy model parameters which include Lamé constants, healthy and pathological atrophy rates, critical biomarker concentration, and biomarker spreading parameters for white matter, gray matter, the hippocampus, ventricles, and cerebrospinal fluid.

	Lamé constants		Atrophy model parameters			Biomarker model parameters	
	λ [kPa]	μ [kPa]	G_h_ [−]	G_c_ [-−]	c^crit^ [−]	d [W/kg m^3^]	α [−]
White matter	64.67	2	0.0015	0.0035	0.5	15	0.09
Gray matter	32.33	1	0.001	0.002	0.5	15	0.09
Hippocampus	32.33	1	0.001	0.002	0.5	15	0.09
Ventricles	29.77	15.34	—	—	—	0	0.09
CSF	7.22	14.43	—	—	—	0	0.09

## Data Availability

The raw data supporting the conclusion of this article will be made available by the authors, without undue reservation.
